# Regulation of hydrogen rich water on strawberry seedlings and root endophytic bacteria under salt stress

**DOI:** 10.3389/fpls.2024.1497362

**Published:** 2024-11-21

**Authors:** Renyuan Wang, Xijia Yang, Yaowei Chi, Xia Zhang, Xianzhong Ma, Dan Zhang, Ting Zhao, Yongfeng Ren, Haiyan Yang, Wenjiang Ding, Shaohua Chu, Pei Zhou

**Affiliations:** ^1^ School of Agriculture and Biology, Shanghai Jiao Tong University, Shanghai, China; ^2^ Key Laboratory of Urban Agriculture, Ministry of Agriculture and Rural Affairs, Shanghai, China; ^3^ Shanghai Yangtze River Delta Eco-Environmental Change and Management Observation and Research Station, Ministry of Science and Technology, Ministry of Education, Shanghai, China; ^4^ Shanghai Key Laboratory of Hydrogen Science and Center of Hydrogen Science, School of Materials Science and Engineering, Shanghai Jiao Tong University, Shanghai, China; ^5^ Yunnan Dali Research Institute of Shanghai Jiao Tong University, Dali, China; ^6^ Inner Mongolia Academy of Agricultural and Animal Husbandry Sciences, Hohhot, China; ^7^ Inner Mongolia Research Institute of Shanghai Jiao Tong University, Hohhot, China

**Keywords:** endophytic bacteria, hydrogen, hydrogen rich water (HRW), salt stress, strawberry

## Abstract

Salt stress could lead to plant growth barriers and crop yield reduction. Strawberries are sensitive to salt stress, and improving salt tolerance is important for strawberry production. This study aimed to explore the potential of hydrogen-rich water (HRW) to enhance salt tolerance in strawberries. Through pot experiments, we investigated how HRW affects plant growth, ion absorption, osmotic stress, oxidative stress, antioxidant enzyme levels, hormone levels, and root endophytic bacteria in strawberry seedlings under salt stress. The results showed that under 100 mM NaCl treatment, 50% and 100% HRW treatments significantly increased strawberry biomass by 0.29 g and 0.54g, respectively, wherein, 100% HRW significantly increased the shoot and root length by 15.34% and 24.49%, respectively. In addition, under salt stress the absorption of K^+^ by strawberry seedlings was increased with the HRW supplement, while the absorption of Na^+^ was reduced. Meanwhile, HRW treatment reduced the transfer of Na^+^ from root to shoot. Furthermore, under salt stress, HRW treatment increased the relative water content (RWC) by 12.35%, decreased the electrolyte leakage rate (EL) by 7.56%. HRW modulated phytohormone levels in strawberry seedlings, thereby alleviating the salt stress on strawberries. Moreover, HRW was found to promote plant growth by altering the diversity of bacteria in strawberry roots and recruiting specific microorganisms, such as *Tistella*. Our findings indicate that HRW could help restore the microecological homeostasis of strawberry seedlings, thus further mitigating salt stress. This study provides a novel perspective on the mechanisms by which HRW alleviates salt stress, thereby enriching the scientific understanding of hydrogen’s applications in agriculture.

## Graphical Abstract

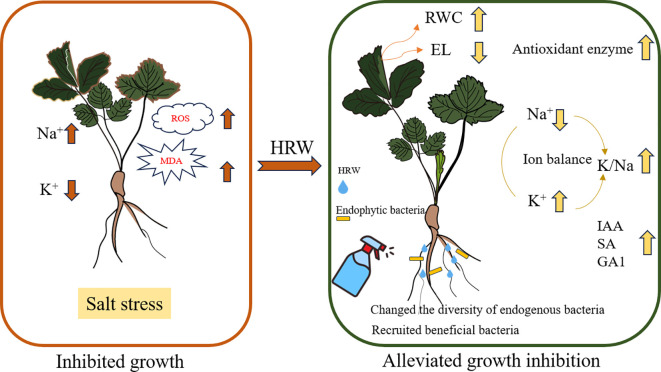

## 1 Introduction

Strawberry (*Fragaria ananassa* duch.) is a perennial herb belonging to genus *Fragaria* in the family Rosaceae, which is widely planted in the world. The world’s strawberry planting area and yield have indeed been increasing, with the planting area reaching 396,400 hectares and the yield reaching 8.89 million tons. This indicates a positive trend in the global strawberry production industry (Lv et al., 2021). China is the world’s largest producer of strawberry, and the planting area and output are also increasing (Su et al., 2022). Additionally, Strawberry has become one of the most popular fruits because of its unique taste and flavor. Strawberries are rich in potential functional compounds such as phenols, phenylpropane compounds, L-ascorbic acid, and other compounds with antioxidant potential. Therefore, they are classified as a functional food source with a variety of health benefits Its demand and availability in the market have greatly increased (Crizel et al., 2020; Moradi et al., 2022). However, strawberries are relatively sensitive to salinity and are vulnerable to salinity. Salt stress will affect the growth and development of strawberries, affecting the absorption of mineral elements, the activity of antioxidant enzymes and the quality of strawberries (Perin et al., 2019; Saidimoradi et al., 2019). Therefore, improving the salt tolerance of strawberries can expand the suitable range of strawberries.

Soil salinity is a major environmental stress that affects agricultural production and environmental health. This phenomenon poses a serious threat to crop growth and also has a profound impact on the physicochemical properties of soil and the stability of ecosystems. In the context of current population growth and climate change, it also poses a threat to food and cultivated land security (Hayat et al., 2020a). Currently, approximately 20% of the global arable land is impacted by salt stress, with around 33% of irrigated land being categorized as affected and deteriorated by salinity (Tefera et al., 2022). Soil salinity will affect the sustainable development of agriculture, ecological environment and food safety. Excessive salinity can alter plant osmotic potential, leading to a decrease in water absorption. Additionally, excessive accumulation of Na^+^ can disrupt ion homeostasis and lead to ion toxicity (Sun et al., 2018; Yang and Guo, 2018b). Na^+^ excessive accumulation effects include inducing the outflow of cell cytosolic K^+^, thereby reducing the cell cytosolic K/Na ratio (Guo et al., 2020). Furthermore, salt stress can cause a sharp increase in H_2_O_2_ levels within plant cells, which might be a crucial signal for salt stress. The excessive accumulation of ROS disrupts cellular metabolism by oxidizing lipids, proteins and nucleic acids, leading to severe oxidative damage (Miller et al., 2010; Xiao and Zhou, 2023). This highlights the urgency of finding solutions to mitigate the adverse effects of soil salinity on crops.

Hydrogen is one of the abundant elements in the universe, and also one of the basic elements of life. Hydrogen is a weak reducing gas with simple structure, which widely exists in the primitive atmosphere. As the most promising energy carrier, hydrogen contributes to the transformation of low-carbon energy and has been widely studied (Zhang et al., 2023). Similarly, numerous studies have shown that hydrogen can significantly improve the oxidative damage of animal cells caused by trauma (Tian et al., 2021; Zhao et al., 2019). In 1964, the botanical effect of H_2_ was first confirmed in Secale cereale. In 2007, Japanese scholars first published hydrogen rich solution, which can be used as a safe and effective exogenous H_2_ source for scientific research (Wang et al., 2024). It is found in nature that microalgae and cyanobacteria can express hydrogenase and reduce protons to gaseous H_2_ (Yacoby et al., 2011). Using hydrogen rich water (HRW) as hydrogen supply mode can improve the tolerance to abiotic and biological stresses, regulate plant growth and development, improve nutritional quality, prolong the shelf life of fruits, and prolong the in bottle-life of fresh cut flowers (Cui et al., 2013; Hu et al., 2018; Su et al., 2019). In addition, the explosion limit of hydrogen in air is 4%-75.6% (Shirvill et al., 2019), and the dissolution limit of hydrogen in water is far less than the explosion concentration. Therefore, it is of great significance to use hydrogen rich water to improve the salt stress resistance of strawberries, alleviate adverse stress, and maintain their normal growth and development, which is a green and sustainable method. In addition, hydrogen can alter the composition of soil bacteria and promote plant growth (Dong et al., 2003). However, the impact of hydrogen on endophytic bacteria within plant roots is still unclear. Therefore, conducting hydrogen research is of great significance for enriching the application of hydrogen in agriculture and improving the theoretical system.

In this study, *Fragaria×ananassa* Duch.’Benihoppe’ was used as the experimental material to screen for salt stress and HRW concentrations that met experimental requirements. The objectives of this study were to: (1) evaluate the effects of hydrogen-rich water (HRW) on the growth of strawberry seedlings under salt stress; (2) investigate the changes in ion absorption, osmotic response, oxidative stress, antioxidant enzyme levels, and hormone levels in strawberry seedlings under salt stress with HRW treatment; and (3) investigate the response of root endophytic bacteria to HRW under salt stress. The study aimed to explore the mechanisms by which HRW alleviates growth inhibition in strawberry seedlings under salt stress, providing a theoretical foundation for the practical application of hydrogen in agricultural environments, thereby contributing to the broader application of hydrogen in enhancing agricultural sustainability.

## 2 Materials and methods

### 2.1 Preparation of HRW

To prepare hydrogen-rich water (HRW) with different saturation levels, pure hydrogen gas (99.99%, v/v) was produced through electrolysis using a QL-300 hydrogen generator from Saikesaisi Hydrogen Energy Co., Ltd., Shandong, China (Su et al., 2019). This hydrogen gas was then bubbled into 4000 mL of deionized water at a flow rate of min^-1^ for a duration of 2h. After the saturation process, the resultant saturated HRW was immediately diluted to create concentrations of 50% and 100% saturation, respectively.

### 2.2 Experimental design

The sources of plant materials and the locations of experiments were provided in the supplemental information. The experiment used a completely randomized block design, with 5 strawberry seedlings planted in each plastic pot (length 44 cm * width 33 cm * height 21 cm). The experiment set up 3 NaCl concentrations: CK (0), Na1 (50 mM), Na2 (100 mM), and 3 hydrogen rich water concentrations: T1(0), T2 (50%), T3 (100%) for a total of 9 treatments (n = 4 each), To mitigate osmotic shock, the plants were watered with a total of 2800 mL of NaCl solution, administered in increments of 400 mL every other day over a period of 2 weeks. Treated with HRW every 2 days. Measured all indicators after 21 days of treatment. The growth parameters of seedlings were determined and frozen at -80°C for other analysis.

### 2.3 Determination of plant physicochemical properties

After 21 days of NaCl stress, the plants were removed from the soilless substrate and washed 3 times with deionized water. Then, divide the plant into shoot and root parts based on the surface of the medium as the boundary. After measuring the length and fresh weight, the separated plants oven-drying at 105°C for 30 minutes, and dry at 70°C for 72 hours until reach a constant weight, measured the dry weight. Dried plant samples were further investigated for determining nutrient elements content and ion accumulation.

### 2.4 Determination MDA, H_2_O_2_, TP and proline

The content of malondialdehyde (MDA) was tested using the thiobarbituric acid (TBA) method at a wavelength of 450, 532 and 530 nm. For the determination of H_2_O_2_, a modified titanium sulfate method was employed, with absorbance measured at 405 nm. The proline content was determined by the acid ninhydrin method, which was measured at a wavelength of 520 nm (Wang et al., 2020; Yang et al., 2023a). The total protein (TP) content in the tissue was measured using a Coomassie Brilliant Blue assay kit, and absorbance was measured at 595 nm using a microplate reader (Nanjing Jiancheng Bioengineering Institute).

### 2.5 Determination of osmotic stress response in strawberry

After washing and wiping the surface of the fresh leaves dry, 2cm x 1cm leaf sections were cut away from the main vein and weighed for their fresh weight (FW). The leaves were then incubated at room temperature (25°C) in 50 ml centrifuge tubes with 20 ml of deionized water for 24 hours, shaken periodically. After incubation, the surface of the leaves was wiped dry again, and they were re-weighed for their turgid weight (TW). Finally, the samples were dried at 70°C for 72 hours, after which they were weighed for their dry weight (DW) (Fairoj et al., 2023). The following formula was used for calculations:


(1) RWC (%)=[(FW–DW)/(TW–DW)]×100

Leaves were washed with deionized water and cut into 2 cm lengths (Lutts et al., 1996). It was placed in a 15 mL centrifuge tube containing 10 mL of deionized water and incubated on a shaking table at 30°C for 6 hours. The conductivity (EC1) of the sample solution was measured using a conductivity meter (Orion Star ™ A212, Thermo Scientific, USA). The centrifuge tubes were boiled in water for 30 minutes, and then measured the conductivity of the sample solution again (EC2).


(2) EL=EC1/EC2

### 2.6 Determination of antioxidant enzymatic activities

For the assessment of oxidative damage and the activities of antioxidant enzymes in strawberry seedlings, leaf tissues weighing 0.5 g were pulverized in an ice bath using 2 mL of cold phosphate buffer solution (PBS, 0.1 M, pH 7.4). The homogenate was then centrifuged at a speed of 4000 rpm for 15 minutes at a temperature of 4°C. Subsequently, the clear liquid above the sediment, or supernatant, was extracted for additional analyses. The oxidative damage in strawberry seedlings was evaluated by measuring the contents of hydrogen peroxide (H_2_O_2_), malondialdehyde (MDA), and free proline. Antioxidative enzymes including SOD, POD, CAT and APX according to the Wang’s method (Wang et al., 2020).

### 2.7 Determination of ion elements in plant

To analyze the presence of K, Na and nutrient elements (K, Na, Ca, Mg) within plant samples, a quantity of 50 mg of plant tissue was subjected to an acid digestion process using a blend of 69% HNO_3_ and 30% HClO_4_ (4:1 v/v) utilizing a graphite digestion unit. This process was continued until the mixture became a clear liquid (Chen et al., 2022). Concentrations of the nutrient elements in the plant tissues were subsequently quantified using an inductively coupled plasma optical emission spectrometry (ICP-OES, Optima 8000, PerkinElmer, USA).

### 2.8 Quantification of phytohormone content

A quantity of 1.5 g of fresh samples was triturated into powder in liquid nitrogen and placed into a glass tube. The extraction of the mixture was carried out by the addition of an isopropanol-water-hydrochloric acid solution, followed by the addition of 8 µL of a 1 µg/mL internal standard. This mixture was then subjected to shaking for 30 min at 4°C. Subsequently, dichloromethane was introduced, and shaking was continued for an additional 30 min under the 4°C. Afterwards, the mixture was centrifuged at 13,000 rpm at 4°C for 5 min, and the organic phase was collected. The organic phase was dried by blowing nitrogen gas under avoidance of light conditions and then re-dissolved in 0.1% v/v formic acid. The solution was subsequently centrifuged at 13,000 rpm at 4°C for 10 min and the supernatant was passed through a 0.22 µm filter membrane. The levels of indole-3-acetic acid (IAA), abscisic acid (ABA), salicylic acid (SA), and gibberellin (GA1) were determined by HPLC-MS/MS (Agilent 1290, USA) (Chi et al., 2022; Wang et al., 2022). For detailed information on the HPLC-MS/MS detection conditions, please refer to the supporting information.

### 2.9 16S rRNA gene sequencing

The collected root samples were placed on ice (to remove impurities adhering to plant roots), the samples were washed with sterile water for 0.5 minutes, then the samples were washed in 75% ethanol for 1 minute, washed with 2% NaClO for 3 minutes, transferred to 75% sterile ethanol for 1 minute, and finally the plant tissue was washed with sterile water for 0.5 minutes (Chi et al., 2023). The nucleic acid extraction of surface disinfected root tissue is carried out at Shanghai Majorbio Biomedical Technology Co., Ltd. Detailed information on specific procedures and analysis can be obtained in the supporting information. The raw data has been uploaded to NCBI (PRJNA1172171).

### 2.10 Statistical analysis

All the data were evaluated as means ± standard errors from three independent biological replications. Analysis of variance (ANOVA) was used for statistical analysis at *p* < 0.05 level, version 22.0 (SPSS, Chicago, IL, USA). Origin 2022b was used for mapping. In order to better understand the correlation between measured parameters, we applied Pearson’s correlation coefficient for analysis.

## 3 Results

### 3.1 Effect of HRW on the growth of strawberry seedlings under salt stress

**Figure 1:**
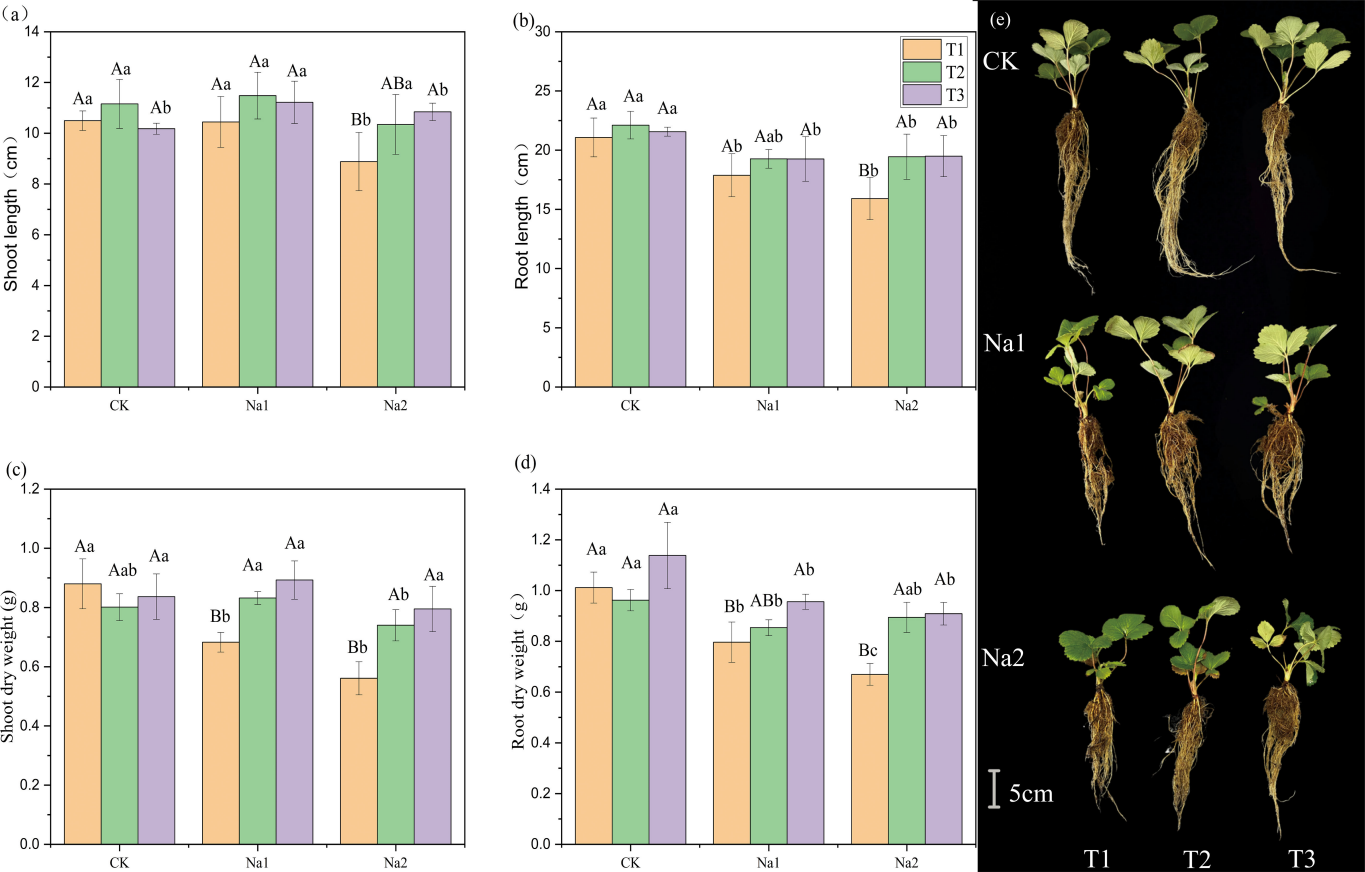
Effects of HRW on length and dry weight of strawberry seedlings shoot and root under salt stress. Different capital letters indicate significant differences between different concentrations of HRW treatments under the same salt treatment. Different lowercase letters indicate significant differences between different concentrations of salt treatments under the same HRW treatment (*p* < 0.05). CK: 0 NaCl, Na1: 50 mM NaCl, Na2: 100 mM NaCl, T1: 0 HRW, T2: 50% HRW, T3: 100% HRW.

To evaluate the potential of HRW in alleviating the growth inhibition of strawberry seedlings under salt stress, the effect of HRW on the phenotype of strawberry seedlings was measured under salt stress. Overall, salt treatment inhibited the growth of strawberry seedlings (**Figure 1**). Under the CK treatment, HRW had no significant effect on growth parameters. Without HRW treatment, the growth parameters of strawberry seedlings decrease with increasing salt concentration. The lengths of shoots and roots are shown in **Figures 1A, B**. On CK and Na1 treatments, different HRW concentration had no significant effect on shoot and root length. However, in the Na2 treatment, compared with T1, T3 significantly increased shoot length and root length by 15.34% and 24.49%, respectively.

HRW had significantly increased the biomass of strawberry seedlings under salt stress (**Figures 1C, D**). Under Na1 treatment, T2 and T3 had significantly increased shoot dry weight by 0.15 g and 0.21 g compared with T1 (0.68 g). In addition, T3 had enhanced root dry weight 0.80 g to 0.96 g (*p*<0.05). Under Na2 treatment, compared with T1, T2 and T3 had significantly increased shoot dry weight by 0.18 g and 0.30 g, and root dry weight by 0.11 g and 0.24 g, respectively. The phenotypic diagram of the plant is shown as **Figure 1E**. In summary, HRW significantly increased the shoot and root lengths and biomass of strawberry seedlings under Na2 treatment, indicating its potential application in alleviating salt stress-induced growth inhibition in strawberry seedlings.

### 3.2 Effect of HRW on shoot oxidative stress in strawberry seedlings under salt stress

**Figure 2:**
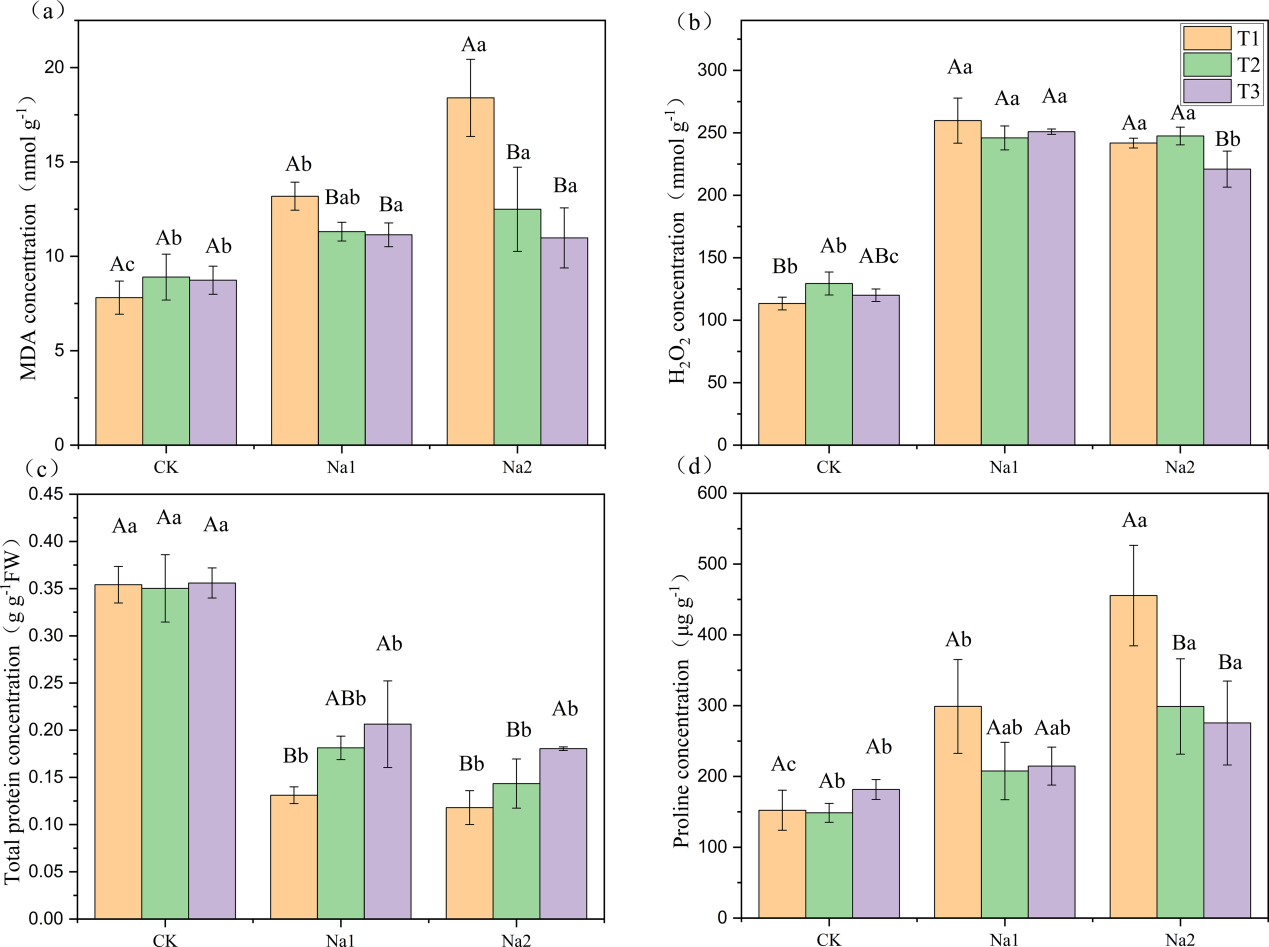
Response of HRW to oxidative stress in strawberry seedlings under salt stress. Different capital letters indicate significant differences between different concentrations of HRW treatments under the same salt treatment (*p* < 0.05). Different lowercase letters indicate significant differences between different concentrations of salt treatments under the same HRW treatment (*p* < 0.05). CK: 0 NaCl, Na1: 50 mM NaCl, Na2: 100 mM NaCl, T1: 0 HRW, T2: 50% HRW, T3: 100% HRW.

As shown in **Figure 2**, MDA and proline concentration increased with increasing salt concentration. Total protein concentration decreased with increasing salt treatment. Meanwhile, salt treatment increased H_2_O_2_ concentration. Compared with T1 (**Figures 2A, D**), T2 and T3 significantly decreased the MDA and proline concentrations under Na2 treatment, proline decreased by 34.40% and 39.52%, respectively. MDA decreased by 32.12% and 40.33%, respectively. In addition, compared with T1 under Na2 treatment, T3 significantly decreased H_2_O_2_ concentration from 241.75 to 220.96 mmol g^-1^, and significantly increased total protein concentration from 1.18 to 1.80 g g^-1^FW (**Figures 2B, C**). However, T2 had no significant effect on H_2_O_2_ concentration and total protein concentration. Overall, HRW significantly reduced the MDA and proline concentrations, decreased H_2_O_2_ levels, and increased total protein concentration in strawberry seedlings under Na2 treatment, indicating its potential benefits in alleviating oxidative stress induced by salt stress.

### 3.3 Effect of HRW on nutrient absorption and ion homeostasis s in strawberry seedlings under salt stress

Based on the indicators mentioned above, we selected the following four groups of treatments for further research, as shown in **Table 1**. In terms of shoot nutrient absorption, Application of HRW had no significant effect on Mg content. Compared with CK, Na treatment was significantly increased Na content by 5.18 time. Similarly, Ca content had increased by 1.11 mg g^-1^ (*p* < 0.05), and K content decreased by 2.14 mg g^-1^ (*p* < 0.05). Compared with Na treatment, HRW treatment significantly reduced the Na and Ca content by 45.05% and 13.22%, respectively. Meanwhile, HRW treatment increased K content by 2.65 mg g^-1^ (*p* < 0.05). K/Na ratio decreased by 80.27% which caused by salt stress, and after HRW treated K/Na ratio increased by 63.96% (*p*<0.05). We also noticed that treatment with HRW alone increased Ca content and K/Na ratio (*p* < 0.05). In root tissue, compared with CK, Na treatment showed a significant increase in Na^+^ by 71.17% and a significant decrease in K^+^ by 6.01%. Na_HRW treatment showed a significant increase of 39.94% in Na^+^. Under salt stress, after applying HRW, K^+^ significantly increased by 12.26%, Na^+^ significantly decreased by 18.25%, Mg^2+^ significantly increased by 4.85%, and K/Na significantly increased by 37.4%. Salt stress significantly enhanced TF_Na_, compared with Na treatment, applying HRW decreased TF_Na_ 32.39% (*p* < 0.05). In conclusion, HRW treatment significantly mitigated salt stress effects on ion uptake in strawberry shoots and roots, enhancing K/Na ratios, thereby improving plant resilience under salinity conditions.

**Table 1:** Nutrient element content in the shoot of strawberry seedlings.

Treatments	Part	K	Na	Ca	Mg	K/Na	TF_K_	TF_Na_
		(mg g^-1^)	(mg g^-1^)	(mg g^-1^)	(mg g^-1^)	(mg g^-1^)		
CK	Shoot	26.38 ± 1.75ab	0.78 ± 0.10 c	12.51 ± 0.93 b	3.79 ± 0.23a	33.76 ± 1.93b	1.57 ± 0.10a	0.24 ± 0.02c
	Root	16.84 ± 0.25b	3.33 ± 0.31 c	12.25 ± 0.22 a	3.15 ± 0.05bc	5.09 ± 0.50b		
HRW	Shoot	27.76 ± 0.55a	0.78 ± 0.01 c	13.62 ± 0.29 a	3.73 ± 0.08a	35.80 ± 0.85a	1.62 ± 0.06a	0.26 ± 0.01c
	Root	17.17 ± 0.40ab	2.98 ± 0.09 d	11.64 ± 0.10 b	3.18 ± 0.03ab	5.76 ± 0.05a		
Na	Shoot	24.24 ± 0.94b	4.04 ± 0.19 a	14.37 ± 0.46 a	3.85 ± 0.10a	6.66 ± 0.04d	1.53 ± 0.05a	0.71 ± 0.03a
	Root	15.83 ± 0.56c	5.70 ± 0.06 a	12.10 ± 0.16 a	3.09 ± 0.05c	2.78 ± 0.11d		
Na_HRW	Shoot	26.89 ± 1.09a	2.22 ± 0.06 b	12.47 ± 0.17 b	3.87 ± 0.01a	10.92 ± 0.39c	1.51 ± 0.04a	0.48 ± 0.03b
	Root	17.77 ± 0.30a	4.66 ± 0.14 b	11.92 ± 0.27 ab	3.24 ± 0.03a	3.81 ± 0.11c		

Different lowercase letters indicate significant differences between different treatments (*p* < 0.05). CK: 0 NaCl+0 HRW, HRW: 0 NaCl+100% HRW, Na: 100 mM NaCl+0 HRW, Na_HRW: 100 mM NaCl+100% HRW.

### 3.4 Effect of HRW on osmotic stress in strawberry seedlings under salt stress

As shown in **Table 2**, compared with CK, Na treatment had significantly decreased RWC by 16.65%, after treated with HRW, RWC increased to 78.44% (*p* < 0.05). Under salt treatment, HRW significantly increased RWC by 12.35%. Compared with CK, Na treatment increased EL by 21.42%, HRW and Na_HRW treatment had decreased EL by 7.31% and 7.56%, respectively (*p* < 0.05). However, compared with Na treatment, Na_HRW treatment increased EL by 13.86% These results indicated that HRW could alleviate adverse effects on water status and electrolyte leakage under salt stress.

**Table 2:** Relative moisture content and electrolyte leakage rate of strawberry leaves.

Group	RWC(%)	EL(%)
CK	82.74 ± 3.63a	40.33 ± 3.87c
HRW	83.16 ± 1.77a	33.02 ± 1.83d
Na	66.09 ± 2.12b	61.75 ± 2.02a
Na_HRW	78.44 ± 2.51a	54.19 ± 5.50b

Different lowercase letters significant differences between different treatments (*p* < 0.05).

CK: 0 NaCl+0 HRW, HRW: 0 NaCl+100% HRW, Na: 100 mM NaCl+0 HRW, Na_HRW: 100 mM NaCl+100% HRW.

### 3.5 Effect of HRW on antioxidant enzyme activity and hormone in strawberry seedlings under salt stress

**Figure 3:**
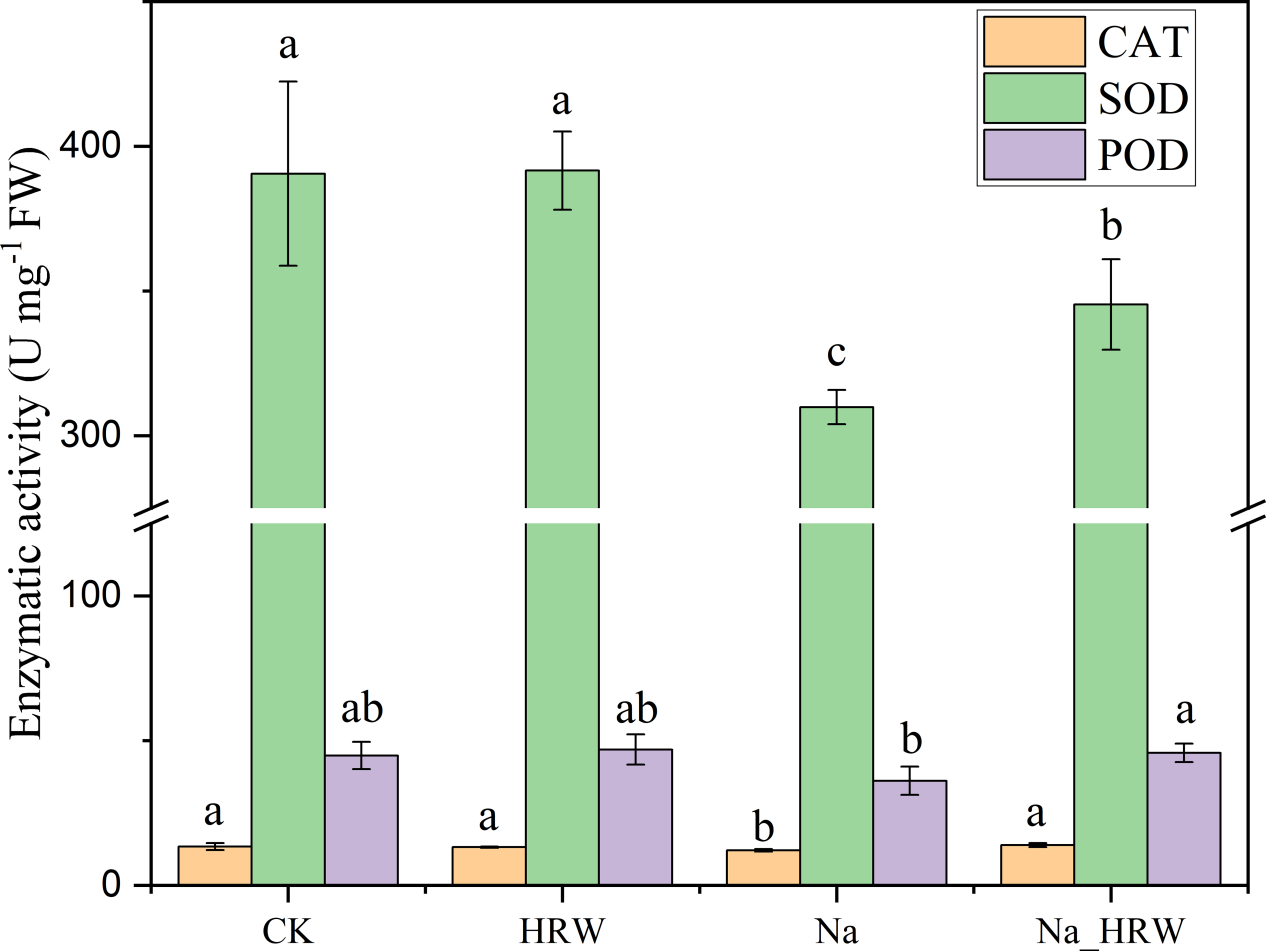
Response of HRW to antioxidant enzyme activity in strawberry seedlings under salt stress. Different lowercase letters indicate significant differences between different treatments (*p* < 0.05). CK: 0 NaCl+0 HRW, HRW: 0 NaCl+100% HRW, Na: 100 mM NaCl+0 HRW, Na_HRW: 100 mM NaCl+100% HRW.

As shown in **Figure 3**, salt stress significantly decreased SOD and CAT activity. Under salt stress, HRW treatment had significantly increased POD, SOD and CAT activity by 1.15, 1.11 and 1.27 times. Unlike POD, SOD and CAT, both salt stress and HRW treatments had no significant impact on POD activity. According to **Table 3**, compared with CK, salt stress significantly reduced the IAA and ABA content in strawberry seedlings. While the SA and GA1 content significantly increased. Compared with Na treatment, Na_HRW treatment significantly increased the IAA, ABA and GA1by 32.23%, 5.75%, and 6.90%, respectively. HRW significantly elevated POD, SOD, and CAT activities in strawberry seedlings under salt stress, and increased the levels of IAA, ABA, and GA1, indicating its role in alleviating the negative effects of salt stress on plant physiological indicators.

**Table 3:** The hormone content in strawberry seedlings.

Group	IAA	ABA	SA	GA1
CK	8.18 ± 0.46a	1.36 ± 0.14b	134.61 ± 2.53c	0.19 ± 0.0013d
HRW	7.31 ± 0.31b	1.65 ± 0.10a	94.97 ± 5.97d	0.25 ± 0.0131c
Na	3.63 ± 0.18d	0.99 ± 0.02c	144.97 ± 1.13b	0.29 ± 0.0073b
Na_HRW	4.80 ± 0.17c	0.73 ± 0.02d	153.30 ± 3.00a	0.31 ± 0.0039a

Different lowercase letters significant differences between different concentrations of salt treatments under the same HRW treatment (*p* < 0.05).

### 3.6 The variation of endosphere bacterial communities in strawberry seedlings

**Figure 4:**
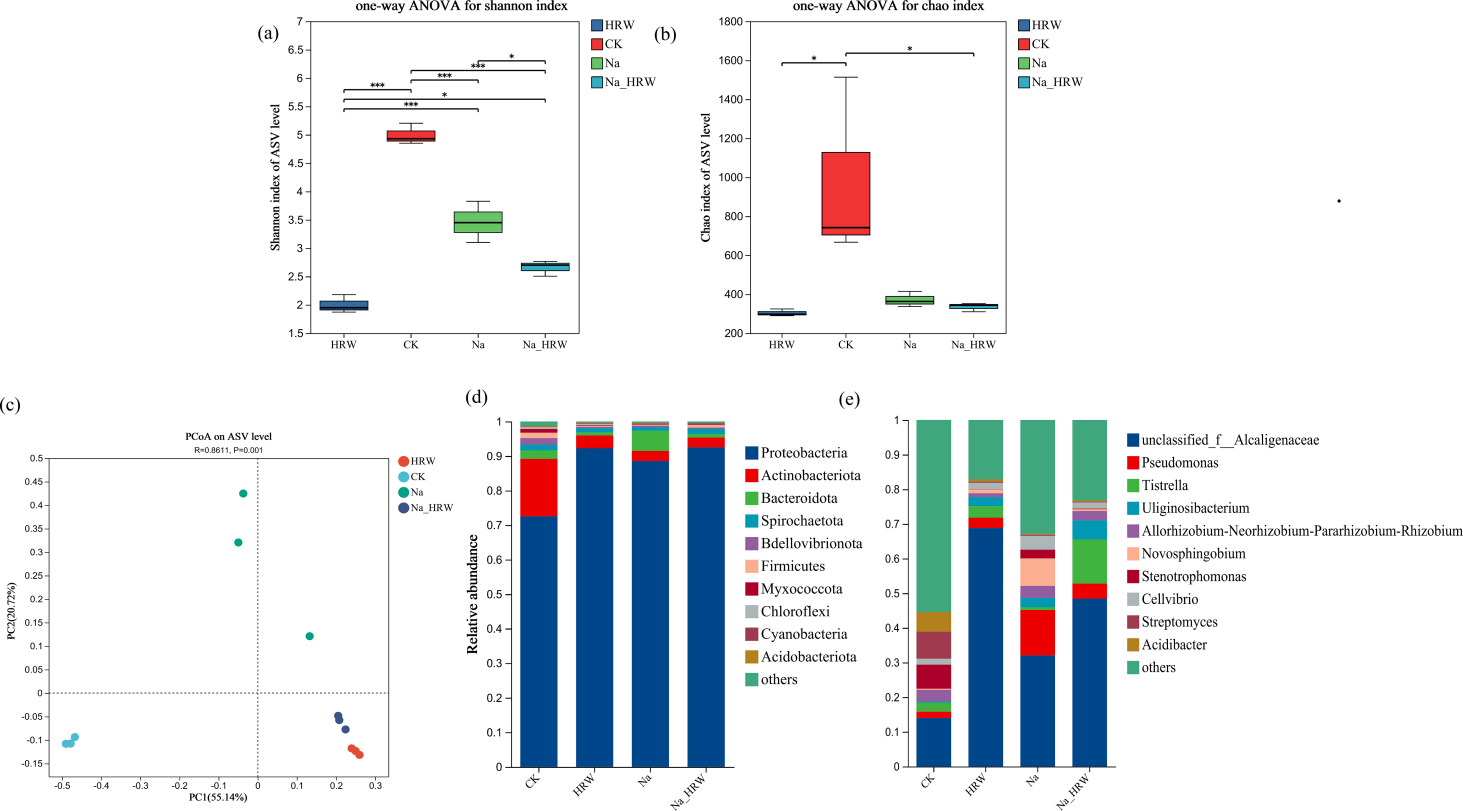
Effect of HRW on the bacterial community in the roots of strawberry seedlings α-diversity **(A, B)**, and β-diversity **(C)**, indicates significant correlations at *p* < 0.05. Effect of HRW inoculation on the composition of bacterial communities in strawberry roots. **(D)** Phylum level composition, **(E)** genus level composition. * indicates *p* < 0.05, *** indicates *p* < 0.001. CK: 0 NaCl+0 HRW, HRW: 0 NaCl+100% HRW, Na: 100 mM NaCl+0 HRW, Na_HRW: 100 mM NaCl+100% HRW.

As shown in **Figures 4A, B**, the Chao index of HRW and Na_HRW was significantly lower than that of CK. However, there was no significant difference between the Na treatment group and the CK and Na HRW treatment groups. The Shannon index showed that all treatments were significantly lower than CK, and the Na_HRW treatment group was significantly lower than the Na treatment group. In addition, Principal Component Analysis (PCA) based on Bray-Curtis showed significant differences between Na-HRW and Na treatments (**Figure 4C**). Further analysis was conducted on the differences in bacterial community composition, according to the results of ASV classification, as shown in **Figure 4D**. At the phylum level, Proteobacteria is the dominant bacterial phylum, followed by Actinobacteriota and Bacteroidota. Compared with CK, the relative abundance of Proteobacteria in the Na treatment group increased from 72.53% to 88.55%, and compared with Na treatment, the Na-HRW treatment increased to 92.50%. At the genus level, the top three genera in relative abundance in the control group were unclassified _ Alcaligenaceae (13.97%), *Streptomyces* (7.81%), and *Stenotrophomonas* (6.93%). HRW treatment significantly increased the relative abundance of unclassified _ Alcaligenaceae to 68.82%, while the abundance of other genera was below 5%. Na treatment, the relative abundance of unclassified _ Alcaligenaceae was 32.04%, *Pseudomonas* was 13.11%, and *Novosphingobium* was 7.96%. Na_HRW enhanced the relative abundance of unclassified _ Alcaligenaceae to 48.43%, while also increasing the abundance of *Tistrella* to 12.73% and *Uliginosibacterium* to 5.47% (**Figure 4E**).

Additionally, Linear Discriminant Analysis Effect Size (LEfSe) analysis was employed to identify the bacteria preferentially recruited by strawberry roots under various treatments, as illustrated in **Supplementary Figure S1**. CK treatment, Actinobacteriota and Rhizobiales emerged as the most significant biomarkers. For the HRW treatment, Alcaligenaceae and Burkholderiales were identified as the predominant biomarkers. In the Na treatment, *Pseudomonas* and Sphingomonadales were highlighted as the key biomarkers, while in the Na_HRW treatment, Proteobacteria and *Tistrella* were identified as the most significant biomarkers (LDA score = 4). We used PICRUSt2 to further investigate whether the use of HRW under salt stress alters the function of bacterial communities. Based on MetaCycle database predictions, results showed that PWY-7111 (pyruvate fermentation to isobutanol), PWY-5101 (L-isoleucine biosynthesis II), VALSYN-PWY (L-valine biosynthesis), ILEUSYN-PWY (L-isoleucine biosynthesis I) which in Na_HRW group was more abundant than Na treatment Endophytic bacteria (**Supplementary Figure S2**). HRW treatment significantly altered the composition of strawberry root bacterial communities under salt stress, increased the relative abundance of Proteobacteria, and identified key biomarkers for each treatment group through LEfSe analysis. Additionally, PICRUSt2 predictions indicated enhanced functional activity of bacterial communities, suggesting that these changes may contribute to the mechanism by which HRW mitigates salt stress in strawberry seedlings.

### 3.7 Correlation between plant growth indicators and parameters

**Figure 5:**
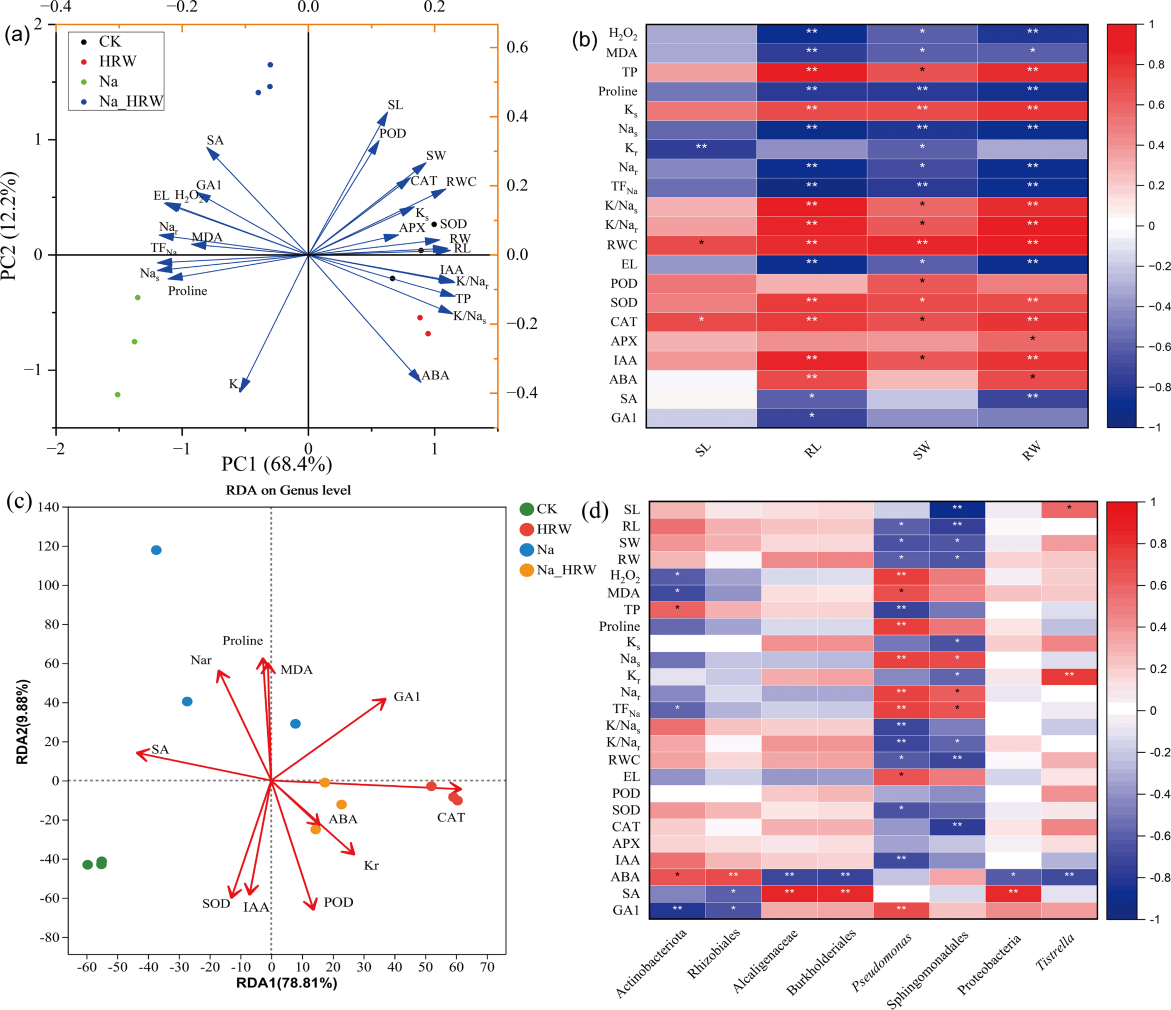
PCA **(A)** and PCC **(B)** were conducted across various treatments, examining the correlation between strawberry physiological attributes. RDA analysis **(C)** relationship between endophytic bacterial communities and key elements in strawberry roots **(C)**. PCC analysis **(D)** between physicochemical factors with significant changes and biomarkers significantly enriched in endophytic bacterial of strawberry root. Plant growth, Na and K content, as well as osmotic and oxidative stress factors, paired with the plant’s antioxidant enzyme activity, employing the Pearson correlation coefficient. Red hues denote positive relationships; blue hues signify negative ones, with deeper coloration corresponding to stronger correlation intensity. * and ** indicates significant differences at the 0.05 and 0.01 probability levels, correspondingly. Ks, K content in shoot part; Kr, K content in root part; Nas, Na content in shoot part; Nar, Na content in root part; SL, length of shoot; RL, length of root; SW, shoot weight; RW, root weight.

As shown in **Figure 5A**, PC1 and PC2 were contributed 80.6% of the variance. PC1 explained 68.4% of total variance, which was correlated with Na_s_, TF_Na_ and RL. PC2 explained 12.2% of total variance, which was correlated with SL, POD and ABA. PCC (**Figure 5B**) showed that the plant biomass (SW and RW) and RL were significantly positively correlated with SOD, POD, RWC and K/Na ratio etc., and negatively correlated with H_2_O_2_, proline, Na content and EL etc. In contrast, SL was positively correlated with RWC and CAT, and negatively correlated with K_r_. RL and RW were positively correlated with IAA, while SA was negatively correlated.

Redundancy analysis (RDA) was used to further characterize the relationship between the bacterial community and key elements in strawberry root endosphere (**Figure 5C**). RDA 1and RDA 2 together explained 88.69% of the variation. Key elements had a major impact on the bacterial community structure (**Supplementary Table S2**). Additionally, based on the selected elements, CK and the Na treatments demonstrated better separation along Axis 2. In contrast, the control group (CK) was more distinctly separated from both the HRW and the Na_HRW treatments along Axis 1. Moreover, Na treatment showed greater separation from the Na_HRW treatment along Axis 2. As illustrated in **Figure 5D**, the biomarkers from Na treatment have a significant positive correlation with Na^+^ content and TF_Na_ and a significant negative correlation with growth indicators. The biomarker Proteobacteria from Na_HRW treatment was significantly positively correlated with SA and negatively correlated with ABA. Moreover, *Tistrella* was significantly positively correlated with SL and Kr and negatively correlated with ABA. These results revealed the relationship between bacterial communities in strawberry roots and key elements, further indicating that HRW treatment improved the growth of strawberry seedlings under salt stress.

## 4 Discussion

Salt stress could inhibit plant growth, in this study, the application of HRW could alleviate the inhibition of salt stress on strawberry seedling growth (**Figure 1**). Previous studies have found that application of HRW can alleviate the inhibition of seedling growth (Zhao et al., 2021), reversed the reduced root length caused by salt stress (Wu et al., 2020), significantly increased fresh weight (Xie et al., 2012).

### 4.1 HRW regulates plant homeostasis and alleviates salt stress

Ion homeostasis is also an important manifestation of salt resistance. Excessive accumulation of Na ions could disrupt ion homeostasis, especially K/Na balance. In this study, Salt treatments increased the Na^+^ content by 5.18 fold, and HRW decreased by 1.82 fold. The K/Na ratio decreased by 5.07-fold under salt stress, while HRW increased the K/Na ratio by 63.96% under salt stress (**Table 2**). These results indicated that HRW positively regulates the potassium sodium ratio and reconstructs ion homeostasis. HRW treatment significantly reduced Na/K ratio under salt stress, caused by the lower Na^+^ accumulation. Similarly, the transcription levels of *SOS1* and *AHA3*, plasma membrane-embedded proteins governing Na^+^ efflux, were measured in seedling roots (Xie et al., 2012). Moreover, during the Na^+^ influx phase, H_2_ alleviated the harmful impact of salinity by enhancing the cytoplasmic K/Na balance in roots exposed to salt (Wu et al., 2020). Sustaining a high potassium-to-sodium ratio when exposed to NaCl is seen as a prime measure of a plant’s salt tolerance and its detoxification mechanisms. Similarly, a high K/Na ratio in plant tissues was previously described as an important criterion for selecting salt tolerant/susceptible varieties (Cristina Paz et al., 2012; Hayat et al., 2020b).

A high concentration of Na in the soil can cause osmotic stress in plants, potentially leading to their dehydration. The main issue is that high levels of Na interfere with water potential, flow properties, and the permeability of cell membranes, thus hindering the soil and roots’ ability to transmit water, subsequently reducing the amount of water that enters the plant (Munns, 2002). RWC (%) is a vital parameter for gauging plant metabolic function and water status under conditions of abiotic stress (Ashraf et al., 2013). In response to salinity stress, plants could activate osmoregulatory mechanisms to preserve cellular volume and sustain turgor pressure (van Zelm et al., 2020).75 mM salinity caused remarkable decrease in strawberry RWC (Zahedi et al., 2019). We found that salt stress significantly reduced leaf RWC and increased EL, indicating that salt stress caused osmotic stress in strawberry seedlings. This is in consistent with the decrease in RWC (%) under NaCl exposure in tomato plant (Tanveer et al., 2020). While HRW significantly increased RWC and decreased EL in strawberry leaves under salt stress. Meanwhile, HRW reduced the content of proline in strawberry leaves under salt stress, which indicated that HRW treatment alleviated osmotic stress caused by salt stress.

ROS are highly active and produced independently in all or most cell compartments, so their levels are controlled to prevent accidental oxidation of cells. This is achieved by balancing the generation, clearance and transport of reactive oxygen species, which will keep the concentration of reactive oxygen species low and control the reactive oxygen signal reaction and its results (Mittler et al., 2022). H_2_O_2_ is a typical ROS in plants. H_2_O_2_ accumulation is a response to a wide range of biotic and abiotic stress (Chen et al., 2021). MDA is a product of membrane lipid peroxidation and is often considered an indicator of damage in plants under stress conditions. Excessive accumulation of MDA and H_2_O_2_ in plants indicates that salt stress induces oxidative stress. Under salt stress, the overproduction of ROS could be detrimental to plant and lead to oxidative stress. Oxidative stress could cause lipid peroxidation, chlorophyll degradation, and damage to the cell membrane, thereby reducing the stability of plant cells (Yang and Guo, 2018a). Salt stress induced H_2_O_2_ increase significantly in both of leaves and roots. Meanwhile, MDA content in the leaves and roots of strawberry was significantly increased under salt stress, which induced oxidative damage in strawberry seedlings (Wu et al., 2020; Xie et al., 2012). Salt stress resulted in a 2.11-fold increase in H_2_O_2_ in strawberry leaves, while HRW treatment significantly reduced the H_2_O_2_ content. Salt stress increased the MDA of strawberry leaves by 1.36 folds, while HRW significantly reduced MDA by 40.32% (**Figure 2**). However, HRW could reconstruct redox homeostasis to alleviate oxidative damage caused by increased ROS levels in strawberry seedlings under salt stress.

Enhancing antioxidant defense capability is considered a key mechanism for plants to cope with various stresses. SOD is considered the first line to clear ROS. It converts superoxide anion radicals into H_2_O_2_. Under normal conditions, POD, APX, and CAT can effectively remove H_2_O_2_. Our results indicate that salt stress reduces the activity of antioxidant enzymes to a certain extent, HRW treatments enhanced the POD, SOD, CAT actives in strawberry seedlings under salt stress (**Figure 3**). c*APX1* may be a target gene involved in H_2_ signal transduction (Xie et al., 2012). Studies have found that HRW can enhance the activities of antioxidant enzymes such as SOD, POD, CAT, and APX, thereby improving salt tolerance in cucumber plants. Additionally, HRW treatment has been shown to upregulate the expression of key genes associated with the activities of *SOD*, *CAT*, and *POD* (Yu et al., 2023). In many plant stress studies, H_2_ might act as a new antioxidant that can significantly reduce the levels of MDA and ROS in plants, and enhance the plant’s antioxidant capacity by markedly increasing the activity of antioxidant enzymes (Cui et al., 2020; Wang et al., 2019; Zhao et al., 2021, 2017). HRW might enable H_2_ readily permeate into the cell membrane thereby influencing gene expression of antioxidant genes (Cui et al., 2013). In summary, based on our results, hydrogen-rich water (HRW) could enhance the antioxidative capacity of strawberry seedlings.

### 4.2 HRW regulates plant hormones to promote strawberry seedlings growth

IAA is an important phytohormone that plays a crucial regulatory role in plant growth and development (Chi et al., 2022). Meanwhile, High concentrations of salt stress have been found to decrease the ability of plant tissues to synthesize IAA, resulting in a decrease in IAA content in plants (Dunlap and Binzel, 1996; Zheng et al., 2020). Therefore, the decrease of IAA content in strawberry seedlings under salt stress may be one of the reasons for the inhibition of their growth and development. However, under salt stress, HRW significantly increased the IAA content in strawberry (**Table 3**). The IAA content exhibited a significant positive correlation with RL, RW, and SW (**Figure 4**). Thereby alleviating the growth limitation of salt stress on strawberry seedlings. Similarly, SA plays a crucial role in defense signal transduction. In this study, under salt stress, the application of HRW significantly increased the SA content in strawberry seedlings (**Table 3**). Previous studies found that SA promoted the production of antioxidant enzymes in cells, thereby alleviating salt stress (Arif et al., 2020). SA could maintain membrane permeability and regulate membrane channels and transport proteins. It mainly regulates plant sodium-potassium balance by limiting the influx of Na^+^ into roots and its transport in shoots, as well as by enhancing the activity of root H^+^ ATPase. Additionally, endogenous SA signaling can also increase the concentration of K^+^ in the aboveground parts, further contributing to the maintenance of ion homeostasis within cells (Fahad et al., 2015; Yang et al., 2023b). GA has been found to play an important role in promoting seed germination, stem elongation, fruit ripening, and enhancing the stress tolerance of plants. Additionally, the application of exogenous GA could also increase the salt tolerance of plants (Hamayun et al., 2010; Wang et al., 2022). Under salt stress, the GA1 content was significantly increased by HRW, indicating its potential involvement in alleviating salt stress in strawberry seedlings (**Table 3**). Therefore, the growth limitation of strawberry seedlings under salt stress might have been mitigated by HRW through the regulation of salt stress phytohormone levels.

### 4.3 HRW modifies the endophytic bacterial community in strawberry roots to attract beneficial companions

The research conducted on the effects of hydrogen evolution during nitrogen fixation and the analysis of rhizosphere microorganisms led to the proposition of the “hydrogen fertilizer theory.” According to this theory, the hydrogen gas liberated by nitrogen-fixing rhizobia, which do not possess hydrogenase enzymes, can facilitate the proliferation of rhizosphere hydrogen-oxidizing bacteria, thereby augmenting plant growth. The successful isolation and purification of hydrogen-oxidizing bacteria provided empirical evidence that these microorganisms are capable of secreting 1-aminocyclopropane-1-carboxylate (ACC) deaminase, a mechanism that has been shown to stimulate plant growth significantly (Dong et al., 2003; Liu et al., 2010). We found that HRW treatment altered the community α-diversity and β-diversity under salt stress (**Figures 4A, B**). Under salt stress, hydrogen treatment was observed to have significantly reduced α-diversity (**Figure 4A**). This phenomenon was also detected in the rhizosphere soil, indicating that hydrogen treatment had decreased the α-diversity of the rhizosphere soil (Li et al., 2018).

Furthermore, hydrogen treatment changed community composition. Hydrogen treatment changed the composition of the root community in strawberry seedlings. Under salt stress, HRW treatment increased the relative abundance of Proteobacteria in the roots of strawberry seedlings, indicating that HRW can enhance the resistance of Proteobacteria to salt stress in strawberry seedlings. Numerous studies have shown that Proteobacteria is a salt tolerant endophytic bacterium identified in plants such as Maize, Potato, Rice Soybean, and Tomato (Yadav et al., 2024). Most proteobacteria in nature were Gram-negative and were found extensively throughout various environments. They played crucial roles in the cycling of nitrogen, sulfur, and carbon within ecosystems (Yue et al., 2022). Proteobacteria significantly contributed to regulate root growth and plant health (Gao et al., 2021). Which might be one of the factors that HRW alleviated growth limitations of strawberry seedlings under salt stress.

Under salt stress, hydrogen treatment increased the relative abundance of unclassified _ Alcaligenaceae, and studies have indicated that certain bacteria within the Alcaligenaceae play a role in promoting plant growth, including the production of IAA, involvement in the nitrogen cycle, and enhancement of disease resistance (Hou et al., 2022; Legrifi et al., 2022). Similarly, the relative abundance of *Tistrella* has also been increased. Limited research has shown that *Tistrella* is a promising microorganism that can be used for bioremediation to remove PAH pollutants from contaminated sites (Zhao et al., 2008). *Tistrella* was capable of degrading chlorpyrifos and breaking it down into non-toxic intermediate metabolites. Research on the effects of intermediate metabolites on mung bean seed germination indicated that, compared to chlorpyrifos, these intermediate metabolites had no toxic impact on seed germination and resulted in increased lengths of shoots and roots (Ahir et al., 2020). Correlation analysis shows that *Tistrella* and unclassified Alcaligenaceae have a positive correlation with strawberry growth, hormone and K ion absorption. Indicated that *Tistrella* and unclassified Alcaligenaceae may have a positive impact on plants under stress. However, further research and analysis were needed to uncover deeper mechanisms.

## 5 Conclusion

Based on the current findings, this study demonstrated that the use of HRW could mitigate excessive Na uptake and shoot transport, enhance the K/Na ratio, thereby increasing RWC, reducing EL, H_2_O_2_, and MDA accumulation. Furthermore, HRW alleviated osmotic stress and boosted antioxidant enzyme activity. HRW also mitigated the growth inhibition of strawberry seedlings caused by salt stress through phytohormone regulation. Notably, HRW altered the diversity of bacteria in strawberry seedling roots, recruiting beneficial bacteria to alleviate salt stress. Multiple mechanisms of HRW in improving plant salt tolerance were demonstrated from multiple perspectives. From the perspective of endophytic bacteria, this discovery not only expands our understanding of the mechanism of HRW action, but also provides a new perspective for improving plant salt tolerance through microbial community regulation. As a green method for enhancing plant salt tolerance, HRW provides valuable insights into the application of hydrogen in agricultural practices. Future research should focus on investigating the long-term effects of HRW on plant growth and further explore and evaluate the broader impacts of HRW across different ecological environments from a molecular ecology perspective.

## Data Availability

The datasets presented in this study can be found in online repositories. The names of the repository/repositories and accession number(s) can be found below: https://www.ncbi.nlm.nih.gov/, PRJNA1172171.

## References

[B1] Ahir U. N. Vyas T. K. Gandhi K. D. Faldu P. R. Patel K. G. (2020). ). *In vitro* efficacy for chlorpyrifos degradation by novel isolate tistrella sp. AUC10 isolated from chlorpyrifos contaminated field. Curr. Microbiol. 77, 2226–2232. doi: 10.1007/s00284-020-01998-1 32361846

[B2] Arif Y. Sami F. Siddiqui H. Bajguz A. Hayat S. (2020). Salicylic acid in relation to other phytohormones in plant: A study towards physiology and signal transduction under challenging environment. Environ. Exp. Bot. 175, 104040. doi: 10.1016/j.envexpbot.2020.104040

[B3] Ashraf M. A. Rasool M. Ali Q. Haider M. Z. Noman A. Azeem M. (2013). Salt-induced perturbation in growth, physiological attributes, activities of antioxidant enzymes and organic solutes in mungbean (Vigna radiata L.) cultivars differing in salinity tolerance. Arch. Agron. Soil Sci. 59, 1695–1712. doi: 10.1080/03650340.2012.758840

[B4] Chen X. Wang J. Hayat K. Zhang D. Zhou P. (2021). Small structures with big impact: Multi-walled carbon nanotubes enhanced remediation efficiency in hyperaccumulator Solanum nigrum L. under cadmium and arsenic stress. Chemosphere 276, 130130. doi: 10.1016/j.chemosphere.2021.130130 33690041

[B5] Chen X. Wang J. You Y. Wang R. Chu S. Chi Y.. (2022). When nanoparticle and microbes meet: The effect of multi-walled carbon nanotubes on microbial community and nutrient cycling in hyperaccumulator system. J. Hazardous Materials 423, 126947. doi: 10.1016/j.jhazmat.2021.126947 34481400

[B6] Chi Y. Ma X. Wu J. Wang R. Zhang X. Chu S.. (2023). Plant growth promoting endophyte promotes cadmium accumulation in *Solanum nigrum* L. by regulating plant homeostasis. J. Hazardous Materials 457, 131866. doi: 10.1016/j.jhazmat.2023.131866 37329596

[B7] Chi Y. You Y. Wang J. Chen X. Chu S. Wang R.. (2022). Two plant growth-promoting bacterial Bacillus strains possess different mechanisms in affecting cadmium uptake and detoxification of Solanum nigrum L. Chemosphere 305, 135488. doi: 10.1016/j.chemosphere.2022.135488 35764116

[B8] Cristina Paz R. Anibal Rocco R. Reinoso H. Bernadina Menendez A. Luis Pieckenstain F. Adolfo Ruiz O. (2012). Comparative study of alkaline, saline, and mixed saline-alkaline stresses with regard to their effects on growth, nutrient accumulation, and root morphology of lotus tenuis. J. Plant Growth Regul. 31, 448–459. doi: 10.1007/s00344-011-9254-4

[B9] Crizel R. L. Perin E. C. Siebeneichler T. J. Borowski J. M. Messias R. S. Rombaldi C. V.. (2020). Abscisic acid and stress induced by salt: Effect on the phenylpropanoid, L-ascorbic acid and abscisic acid metabolism of strawberry fruits. Plant Physiol. Biochem. 152, 211–220. doi: 10.1016/j.plaphy.2020.05.003 32428822

[B10] Cui W. Gao C. Fang P. Lin G. Shen W. (2013). Alleviation of cadmium toxicity in Medicago sativa by hydrogen-rich water. J. Hazardous Materials 260, 715–724. doi: 10.1016/j.jhazmat.2013.06.032 23846121

[B11] Cui W. Yao P. Pan J. Dai C. Cao H. Chen Z.. (2020). Transcriptome analysis reveals insight into molecular hydrogen-induced cadmium tolerance in alfalfa: the prominent role of sulfur and (homo)glutathione metabolism. BMC Plant Biol. 20, 58. doi: 10.1186/s12870-020-2272-2 32019510 PMC7001311

[B12] Dong Z. Wu L. Kettlewell B. Caldwell C. D. Layzell D. B. (2003). Hydrogen fertilization of soils - is this a benefit of legumes in rotation? Plant Cell Environ. 26, 1875–1879. doi: 10.1046/j.1365-3040.2003.01103.x

[B13] Dunlap J. R. Binzel M. L. (1996). NaCl reduces indole-3-acetic acid levels in the roots of tomato plants independent of stress-induced abscisic acid. Plant Physiol. 112, 379–384. doi: 10.1104/pp.112.1.379 12226396 PMC157959

[B14] Fahad S. Hussain S. Matloob A. Khan F. A. Khaliq A. Saud S.. (2015). Phytohormones and plant responses to salinity stress: a review. Plant Growth Regul. 75, 391–404. doi: 10.1007/s10725-014-0013-y

[B15] Fairoj S. A. Islam M. M. Islam M. A. Zaman E. Momtaz M. B. Hossain M. S.. (2023). Salicylic acid improves agro-morphology, yield and ion accumulation of two wheat (*Triticum aestivum* L.) genotypes by ameliorating the impact of salt stress. Agronomy-Basel 13 (1), 25. doi: 10.3390/agronomy13010025

[B16] Gao Y. Cui J. Ren G. Wei S. Yang P. Yin C.. (2021). Changes in the root-associated bacteria of sorghum are driven by the combined effects of salt and sorghum development. Environ. Microbiome 16, 14. doi: 10.1186/s40793-021-00383-0 34380546 PMC8356455

[B17] Guo Q. Meng L. Han J. Mao P. Tian X. Zheng M.. (2020). SOS1 is a key systemic regulator of salt secretion and K+/Na+ homeostasis in the recretohalophyte Karelinia caspia. Environ. Exp. Bot. 177, 104098. doi: 10.1016/j.envexpbot.2020.104098

[B18] Hamayun M. Khan S. A. Khan A. L. Shin J.-H. Ahmad B. Shin D.-H.. (2010). Exogenous gibberellic acid reprograms soybean to higher growth and salt stress tolerance. J. Agric. Food Chem. 58, 7226–7232. doi: 10.1021/jf101221t 20509656

[B19] Hayat K. Bundschuh J. Jan F. Menhas S. Hayat S. Haq F.. (2020a). Combating soil salinity with combining saline agriculture and phytomanagement with salt-accumulating plants. Crit. Rev. Environ. Sci. Technol. 50, 1085–1115. doi: 10.1080/10643389.2019.1646087

[B20] Hayat K. Zhou Y. Menhas S. Bundschuh J. Hayat S. Ullah A.. (2020b). Pennisetum giganteum: An emerging salt accumulating/tolerant non-conventional crop for sustainable saline agriculture and simultaneous phytoremediation. Environ. pollut. 265, 114876. doi: 10.1016/j.envpol.2020.114876 32512425

[B21] Hou T.-T. Miao L.-L. Peng J.-S. Ma L. Huang Q. Liu Y.. (2022). Dirammox is widely distributed and dependently evolved in *alcaligenes* and is important to nitrogen cycle. Front. Microbiol. 13. doi: 10.3389/fmicb.2022.864053 PMC9136411 35633697

[B22] Hu H. Zhao S. Li P. Shen W. (2018). Hydrogen gas prolongs the shelf life of kiwifruit by decreasing ethylene biosynthesis. Postharvest Biol. Technol. 135, 123–130. doi: 10.1016/j.postharvbio.2017.09.008

[B23] Legrifi I. Al Figuigui J. El Hamss H. Lazraq A. Belabess Z. Tahiri A.. (2022). Potential for Biological Control of *Pythium schmitthenneri* Root Rot Disease of Olive Trees (*Olea europaea* L.) by Antagonistic Bacteria. Microorganisms 10, 1635. doi: 10.3390/microorganisms10081635 36014053 PMC9412840

[B24] Li Z. Liu X. Liu R. Li L. Wang L. Wang W. (2018). Insight into Bacterial Community Diversity and Monthly Fluctuations of *Medicago sativa* Rhizosphere Soil in Response to Hydrogen Gas Using Illumina High-Throughput Sequencing. Curr. Microbiol. 75, 1626–1633. doi: 10.1007/s00284-018-1569-y 30206668

[B25] Liu H. Wang W. Cao G. Tang M. (2010). Effect of hydrogen on microbial population and enzyme activity in *robinia pseudoacacia* rhizosphere soil. Chin. J. Appl. Environ. Biol. 16, 515–518. doi: 10.3724/sp.J.1145.2010.00515

[B26] Lutts S. Kinet J. M. Bouharmont J. (1996). NaCl-induced senescence in leaves of rice (Oryza sativa L) cultivars differing in salinity resistance. Ann. Bot. 78, 389–398. doi: 10.1006/anbo.1996.0134

[B27] Lv Y. Li Y. Liu X. Xu K. (2021). Effect of soil sulfamethoxazole on strawberry (Fragaria ananassa): Growth, health risks and silicon mitigation*. Environ. pollut. 286, 117321. doi: 10.1016/j.envpol.2021.117321 33975211

[B28] Miller G. Suzuki N. Ciftci-Yilmaz S. Mittler R. (2010). Reactive oxygen species homeostasis and signalling during drought and salinity stresses. Plant Cell Environ. 33, 453–467. doi: 10.1111/j.1365-3040.2009.02041.x 19712065

[B29] Mittler R. Zandalinas S. I. Fichman Y. Van Breusegem F. (2022). Reactive oxygen species signalling in plant stress responses. Nat. Rev. Mol. Cell Biol. 23, 663–679. doi: 10.1038/s41580-022-00499-2 35760900

[B30] Moradi P. Vafaee Y. Mozafari A. A. Tahir N. A.-R. (2022). Silicon nanoparticles and methyl jasmonate improve physiological response and increase expression of stress-related genes in strawberry cv. Paros under salinity stress. Silicon 14, 10559–10569. doi: 10.1007/s12633-022-01791-8

[B31] Munns R. (2002). Comparative physiology of salt and water stress. Plant Cell Environ. 25, 239–250. doi: 10.1046/j.0016-8025.2001.00808.x 11841667

[B32] Perin E. C. Messias R. d. S. Borowski J. M. Crizel R. L. Schott I. B.. (2019). ABA-dependent salt and drought stress improve strawberry fruit quality. Food Chem. 271, 516–526. doi: 10.1016/j.foodchem.2018.07.213 30236710

[B33] Saidimoradi D. Ghaderia N. Javadi T. (2019). Salinity stress mitigation by humic acid application in strawberry (Fragaria x ananassa Duch.). Scientia Hortic. 256, 108594. doi: 10.1016/j.scienta.2019.108594

[B34] Shirvill L. C. Roberts T. A. Royle M. Willoughby D. B. Sathiah P. (2019). Experimental study of hydrogen explosion in repeated pipe congestion - Part 1: Effects of increase in congestion. Int. J. Hydrogen Energy 44, 9466–9483. doi: 10.1016/j.ijhydene.2018.04.193

[B35] Su D. Chen S. Zhou W. Yang J. Luo Z. Zhang Z.. (2022). Comparative analysis of the microbial community structures between healthy and anthracnose-infected strawberry rhizosphere soils using illumina sequencing technology in yunnan province, southwest of China. Front. Microbiol. 13. doi: 10.3389/fmicb.2022.881450 PMC9149601 35651487

[B36] Su J. Nie Y. Zhao G. Cheng D. Wang R. Chen J.. (2019). Endogenous hydrogen gas delays petal senescence and extends the vase life of lisianthus cut flowers. Postharvest Biol. Technol. 147, 148–155. doi: 10.1016/j.postharvbio.2018.09.018

[B37] Sun M.-H. Ma Q.-J. Hu D.-G. Zhu X.-P. You C.-X. Shu H.-R.. (2018). The glucose sensor mdHXK1 phosphorylates a tonoplast na+/H+ Exchanger to improve salt tolerance. Plant Physiol. 176, 2977–2990. doi: 10.1104/pp.17.01472 29440593 PMC5884615

[B38] Tanveer K. Gilani S. Hussain Z. Ishaq R. Adeel M. Ilyas N. (2020). Effect of salt stress on tomato plant and the role of calcium. J. Plant Nutr. 43, 28–35. doi: 10.1080/01904167.2019.1659324

[B39] Tefera B. B. Bayabil H. K. Tong Z. Teshome F. T. Wenbo P. Li Y. C.. (2022). Using liquefied biomass hydrogel to mitigate salinity in salt-affected soils. Chemosphere 309, 136480. doi: 10.1016/j.chemosphere.2022.136480 36162515

[B40] Tian Y. Zhang Y. Wang Y. Chen Y. Fan W. Zhou J.. (2021). Hydrogen, a novel therapeutic molecule, regulates oxidative stress, inflammation, and apoptosis. Front. Physiol. 12. doi: 10.3389/fphys.2021.789507 PMC8721893 34987419

[B41] van Zelm E. Zhang Y. Testerink C. (2020). Salt tolerance mechanisms of plants. Annu. Rev. Plant Biol. 71, 403–433.doi: 10.1146/annurev-arplant-050718-100005 32167791

[B42] Wang B. Bian B. Wang C. Li C. Fang H. Zhang J.. (2019). Hydrogen gas promotes the adventitious rooting in cucumber under cadmium stress. PloS One 14, e0212639. doi: 10.1371/journal.pone.0212639 30785953 PMC6382157

[B43] Wang J. Chen X. Chi Y. Chu S. Hayat K. Zhi Y.. (2020). Optimization of NPK fertilization combined with phytoremediation of cadmium contaminated soil by orthogonal experiment. Ecotoxicology Environ. Saf. 189, 109997. doi: 10.1016/j.ecoenv.2019.109997 31812023

[B44] Wang J. Lv P. Yan D. Zhang Z. Xu X. Wang T.. (2022). Exogenous Melatonin Improves Seed Germination of Wheat (*Triticum aestivum* L.) under Salt Stress. Int. J. Mol. Sci. 23, 8436. doi: 10.3390/ijms23158436 35955571 PMC9368970

[B45] Wang R. Yang X. Chen X. Zhang X. Chi Y. Zhang D.. (2024). A critical review for hydrogen application in agriculture: Recent advances and perspectives. Crit. Rev. Environ. Sci. Technol. 54, 222–238. doi: 10.1080/10643389.2023.2232253

[B46] Wu Q. Su N. Shabala L. Huang L. Yu M. Shabala S. (2020). Understanding the mechanistic basis of ameliorating effects of hydrogen rich water on salinity tolerance in barley. Environ. Exp. Bot. 177, 104136. doi: 10.1016/j.envexpbot.2020.104136

[B47] Xiao F. Zhou H. (2023). Plant salt response: Perception, signaling, and tolerance. Front. Plant Sci. 13. doi: 10.3389/fpls.2022.1053699 PMC9854262 36684765

[B48] Xie Y. Mao Y. Lai D. Zhang W. Shen W. (2012). H-2 enhances arabidopsis salt tolerance by manipulating ZAT10/12-mediated antioxidant defence and controlling sodium exclusion. PloS One 7, e49800. doi: 10.1371/journal.pone.0049800 23185443 PMC3504229

[B49] Yacoby I. Pochekailov S. Toporik H. Ghirardi M. L. King P. W. Zhang S. (2011). Photosynthetic electron partitioning between FeFe -hydrogenase and ferredoxin:NADP(+)-oxidoreductase (FNR) enzymes *in vitro* . Proc. Natl. Acad. Sci. United States America 108, 9396–9401. doi: 10.1073/pnas.1103659108 PMC3111258 21606330

[B50] Yadav J. Srivastva A. K. Singh R. (2024). Diversity of halotolerant endophytes from wheat (*Triticum aestivum*) and their response to mitigate salt stress in plants. Biocatalysis Agric. Biotechnol. 56, 103000. doi: 10.1016/j.bcab.2023.103000

[B51] Yang Y. Guo Y. (2018a). Elucidating the molecular mechanisms mediating plant salt-stress responses. New Phytol. 217, 523–539. doi: 10.1111/nph.14920 29205383

[B52] Yang Y. Guo Y. (2018b). Unraveling salt stress signaling in plants. J. Integr. Plant Biol. 60, 796–804. doi: 10.1111/jipb.12689 29905393

[B53] Yang F. Liu Y. Zhang X. Liu X. Wang G. Jing X.. (2023a). Oxidative post-translational modification of catalase confers salt stress acclimatization by regulating H_2_O_2_ homeostasis in *Malus hupehensis* . J. Plant Physiol. 287, 154037. doi: 10.1016/j.jplph.2023.154037 37354701

[B54] Yang W. Zhou Z. Chu Z. (2023b). Emerging roles of salicylic acid in plant saline stress tolerance. Int. J. Mol. Sci. 24, 3388. doi: 10.3390/ijms24043388 36834798 PMC9961897

[B55] Yu Y. Zhang H. Xing H. Cui N. Liu X. Meng X.. (2023). Regulation of growth and salt resistance in cucumber seedlings by hydrogen-rich water. J. Plant Growth Regul. 42, 134–153. doi: 10.1007/s00344-021-10536-7

[B56] Yue H. Zhao L. Yang D. Zhang M. Wu J. Zhao Z.. (2022). Comparative analysis of the endophytic bacterial diversity of *populus euphratica* olive. in environments of different salinity intensities. Microbiol. Spectr. 10, e00500–e00522. doi: 10.1128/spectrum.00500-22 35587636 PMC9241684

[B57] Zahedi S. M. Abdelrahman M. Hosseini M. S. Hoveizeh N. F. Lam-Son Phan T. (2019). Alleviation of the effect of salinity on growth and yield of strawberry by foliar spray of selenium-nanoparticles. Environ. pollut. 253, 246–258. doi: 10.1016/j.envpol.2019.04.078 31319241

[B58] Zhang T. Uratani J. Huang Y. Xu L. Griffiths S. Ding Y. (2023). Hydrogen liquefaction and storage: Recent progress and perspectives. Renewable Sustain. Energy Rev. 176, 113204. doi: 10.1016/j.rser.2023.113204

[B59] Zhao Y.-S. An J.-R. Yang S. Guan P. Yu F.-Y. Li W.. (2019). Hydrogen and oxygen mixture to improve cardiac dysfunction and myocardial pathological changes induced by intermittent hypoxia in rats. Oxid. Med. Cell. Longevity 2019, 7415212. doi: 10.1155/2019/7415212 PMC6431505 30984338

[B60] Zhao X. Chen Q. Wang Y. Shen Z. Shen W. Xu X. (2017). Hydrogen-rich water induces aluminum tolerance in maize seedlings by enhancing antioxidant capacities and nutrient homeostasis. Ecotoxicology Environ. Saf. 144, 369–379. doi: 10.1016/j.ecoenv.2017.06.045 28647604

[B61] Zhao G. Cheng P. Zhang T. Abdalmegeed D. Xu S. Shen W. (2021). Hydrogen-rich water prepared by ammonia borane can enhance rapeseed (Brassica napus L.) seedlings tolerance against salinity, drought or cadmium. Ecotoxicology Environ. Saf. 224, 112640. doi: 10.1016/j.ecoenv.2021.112640 34392154

[B62] Zhao H.-P. Wang L. Ren J.-R. Li Z. Li M. Gao H.-W. (2008). Isolation and characterization of phenanthrene-degrading strains *Sphingomonas* sp ZP1 and *Tistrella* sp ZP5. J. Hazardous Materials 152, 1293–1300. doi: 10.1016/j.jhazmat.2007.08.008 17850962

[B63] Zheng L. Ma H. Jiao Q. Ma C. Wang P. (2020). Phytohormones: important participators in plant salt tolerance. Int. J. Agric. Biol. 24, 319–332. doi: 10.17957/ijab/15.1441

