# Supplementary material for: Regulation of hydrogen rich water on strawberry seedlings and root endophytic bacteria under salt stress
Source: Front Plant Sci. 2024 Nov 21;15:1497362. doi: 10.3389/fpls.2024.1497362 (PMC11617194; doi:10.3389/fpls.2024.1497362)
Supplement: Supplementary file 1 [file DataSheet1.docx]

**Supplementary Information**

**Regulation of hydrogen rich water on strawberry seedlings and root endophytic bacteria under salt stress**

Renyuan Wang ^a,b,c,d^, , Xijia Yang ^a,b,c^, Yaowei Chi ^a,b,c^ Xia Zhang ^a,b,c^, Xianzhong Ma^a,b,c^, Dan Zhang ^a,b,c,d,e^, Ting Zhao^e^, Yongfeng Ren^g^, Haiyan Yang^d^, Wenjiang Ding^d^, , Shaohua Chu* ^a,b,c,d,e,f^, Pei Zhou* ^a,b,c,d,e,f^.

a School of Agriculture and Biology, Shanghai Jiaotong University, Shanghai 200240, China;

b Key Laboratory of Urban Agriculture, Ministry of Agriculture and Rural Affairs, Shanghai 200240, China;

c Shanghai Yangtze River Delta Eco-Environmental Change and Management Observation and Research Station, Ministry of Science and Technology, Ministry of Education, Shanghai 200240, China;

d Shanghai Key Laboratory of Hydrogen Science and Center of Hydrogen Science, School of Materials Science and Engineering, Shanghai Jiao Tong University, Shanghai 200240, China;

e Yunnan Dali Research Institute of Shanghai Jiaotong University; Dali 671006, China;

f Inner Mongolia Research Institute of Shanghai Jiao Tong University, Hohhot, 010052, China;

g Inner Mongolia Academy of Agricultural and Animal Husbandry Sciences, Hohhot, 010031, China.

＊Corresponding authors. Email addresses: peizhousjtu@163.com (P. Zhou), chushyt@sjtu.edu.cn (S. Chu).

**Materials and methods**

After cultured the seedlings with substrate for one week, 5 leaves seedlings were chosen to a plastic pot (44*33*21cm) with 25 L soilless substrate (Table S1). The experiment was conducted in a greenhouse at the College of Agriculture and Biology, Shanghai Jiao Tong University, Shanghai, China (31°11′N,121°36′E). Strawberry seedlings used in the experiment were obtained from tissue culture seedlings of SHANGHAI WELLS SEED CO., LTD.

Extract the total DNA of the microbial community according to the E.Z.N.A.® soil DNA kit (Omega Bio-tek, Norcross, GA, U.S.), use 1% agarose gel electrophoresis to detect the DNA extraction quality, and use NanoDrop 2000 to determine the DNA concentration and purity; The nested PCR method was used for amplification. In the first round, primers 799F (5 '- ACMGGATTATACCKG3') and 1392R (5 '- ACMGGATTAGATGTGTRC-3') were used. In the second round, primers 799F (5 '- ACMGGATTATACCKG-3') and 1193R (5 '- ACMGGATTATACCC-3') were used to amplify the V5-V7 regions of the 16S rRNA gene. Use the AxyPrep DNA Gel Extraction Kit (Axygen Biosciences, Union City, CA, USA) to purify the recovered product, 2% agarose gel electrophoresis detection, and use Quantus™ Fluorometer (Promega, USA) was used to quantify the recovered product. Use the NEXTFLEX Rapid DNA Seq Kit for library construction. Use Illumina Miseq PE300 platform for sequencing^[1, 2]^.

Tbale S1 The basic properties of soilless substrate

| Organic mater | pH | EC | TDS | Pb (mg Kg^-1^) | Cr (mg Kg^-1^) | Hg | As (mg Kg^-1^) | Cd |
| --- | --- | --- | --- | --- | --- | --- | --- | --- |
| 47.83% | 5.94 | 0.478 mS/cm | 234.6 ppm | Non | 16.96 | Non | 2.36 | Non |

Table S2 RDA analysis results

| Items | RDA1 | RDA2 | r^2^ | p_value |
| --- | --- | --- | --- | --- |
| Proline | -0.0432 | 0.9991 | 0.6054 | 0.014 |
| MDA | -0.0179 | 0.9998 | 0.5569 | 0.024 |
| CAT | 0.9974 | -0.0714 | 0.6012 | 0.012 |
| POD | 0.2036 | -0.979 | 0.7093 | 0.004 |
| SOD | -0.2106 | -0.9776 | 0.5834 | 0.014 |
| K+ | 0.5804 | -0.8143 | 0.3305 | 0.157 |
| Na+ | -0.2902 | 0.957 | 0.5333 | 0.026 |
| IAA | -0.1194 | -0.9928 | 0.5336 | 0.026 |
| ABA | 0.5642 | -0.8257 | 0.1147 | 0.596 |
| SA | -0.9512 | 0.3087 | 0.3257 | 0.165 |
| GA1 | 0.6643 | 0.7475 | 0.4851 | 0.049 |

The r^2^ value represents the coefficient of determination between environmental factors and species distribution (ranging from 0 to 1), and the smaller the value, the smaller the impact of the environmental factor on species distribution; The *p*-values represent the significance test values of the correlation, with *p* < 0.05 indicating significant correlation.


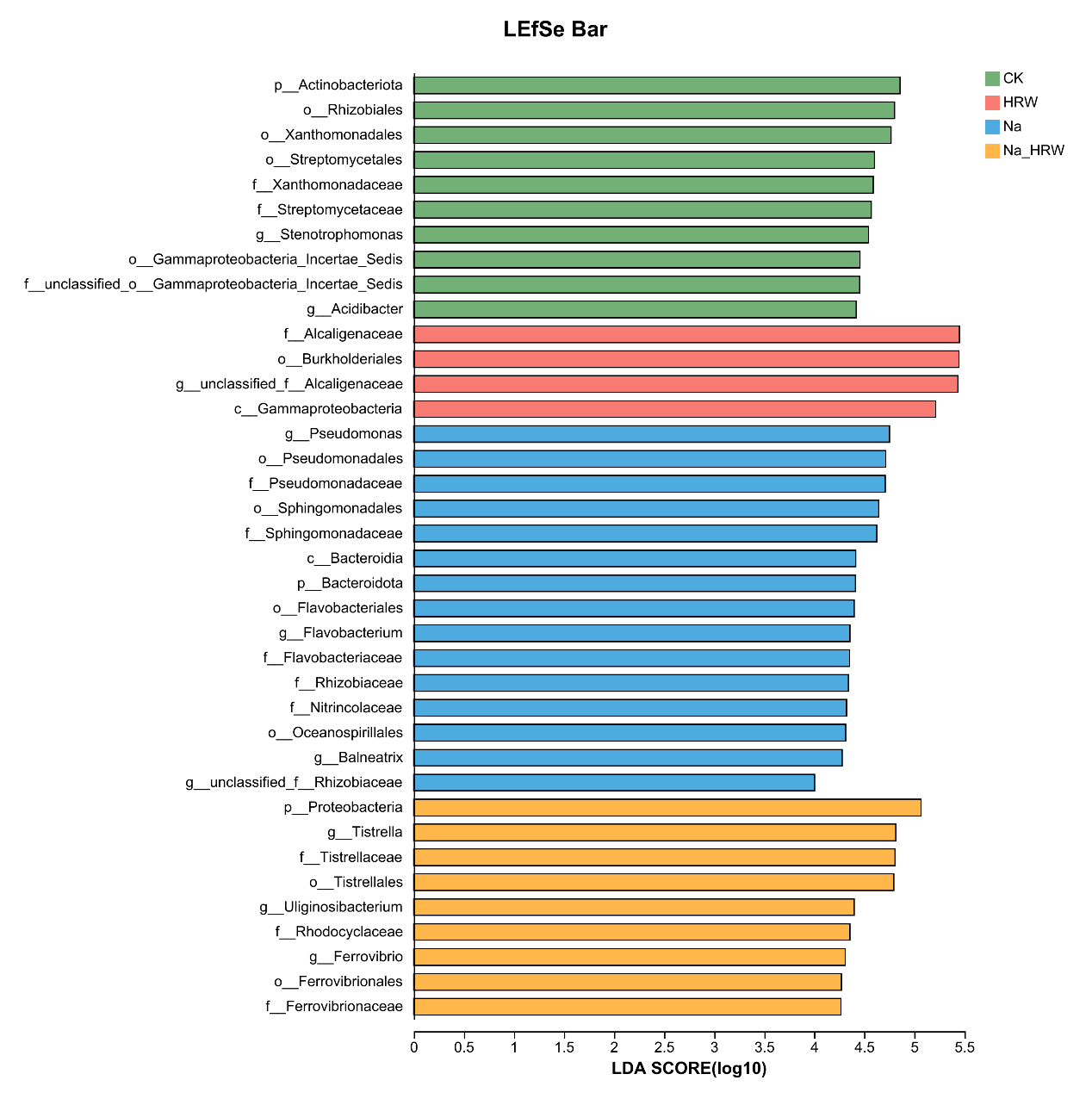


Fig S1. LDA effect size analysis of the root endosphere bacterial community.

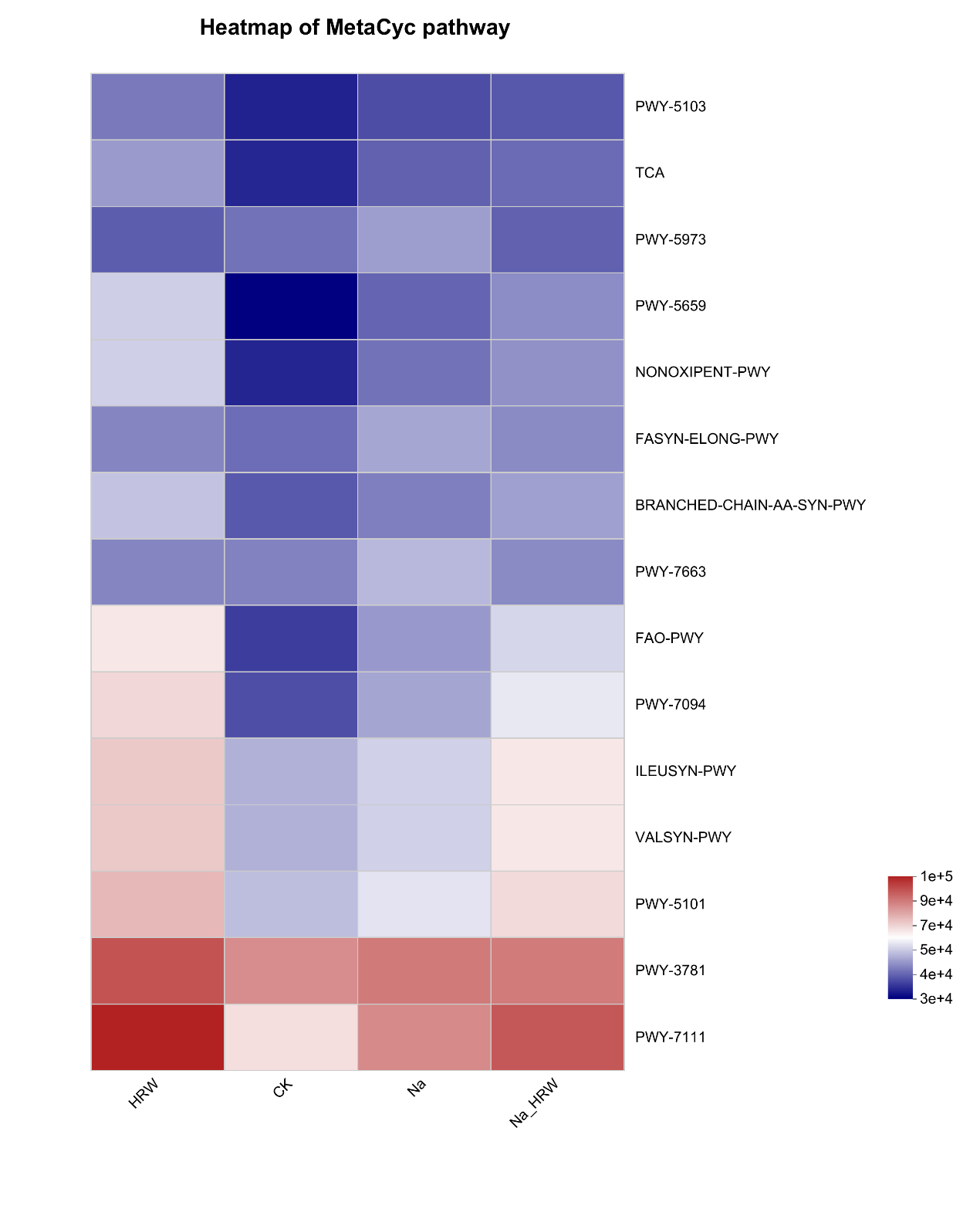
Fig S2 Functional profiling of endosphere bacterial communities under different treatment.

**Reference**

[1] Wang Y, Zhang W, Ding C, et al. Endophytic Communities of Transgenic Poplar Were Determined by the Environment and Niche Rather Than by Transgenic Events [J]. Frontiers in Microbiology, 2019, 10

[2] Wang L, Lin H, Dong Y, et al. Effects of endophytes inoculation on rhizosphere and endosphere microecology of Indian mustard (<i>Brassica</i> <i>juncea</i>) grown in vanadium-contaminated soil and its enhancement on phytoremediation [J]. Chemosphere, 2020, 240
